# Culturally sensitive psychotherapy—technique or attitude?

**DOI:** 10.3389/fpsyg.2025.1599855

**Published:** 2025-10-02

**Authors:** Markus Stingl, Bernd Hanewald

**Affiliations:** Center for Psychiatry and Psychotherapy, Justus-Liebig-University Giessen, Giessen, Germany

**Keywords:** culture, psychotherapy, migration, mental health, intercultural awareness and competence

## Abstract

Culturally sensitive psychotherapy is essential in increasingly diverse societies, where cultural, religious, and linguistic differences shape how distress is experienced and communicated. This article conceptualizes culturally sensitive psychotherapy not only as a set of techniques, but as a reflective professional attitude. Drawing on models of intercultural competence and clinical examples, the article explores how culture affects the expression of symptoms, help-seeking behavior, and therapeutic relationships, especially among migrants. Integrating the cultural contexts enhances diagnostic accuracy, therapeutic alliance, and treatment outcomes. The approach balances awareness of cultural influence with the risk of stereotyping, urging clinicians to adopt a self-reflective stance. Culturally sensitive psychotherapy thus fosters effective and respectful care across diverse populations.

## Introduction

In the context of globalization, increasing forced displacement, and persistent disparities in access and outcomes of mental health care, the relevance of culturally sensitive psychotherapy has become more urgent than ever. Across Europe and around the world, societies are becoming more diverse due to complex patterns of migration, including labor mobility, asylum seeking, and involuntary displacement caused by war, persecution, and climate change. According to UN High Commissioner for Refugees (UNHCR) (2025), more than 117 million people are currently forcibly displaced worldwide—a number unprecedented in recent history. This demographic transformation presents both ethical and clinical challenges for mental health care systems. Migrants and refugees often face elevated rates of mental health problems, including depression, anxiety, PTSD, and somatic symptom disorders, frequently shaped by trauma, loss, and post-migration stressors. At the same time, structural and cultural barriers—including linguistic obstacles, stigma, and divergent health beliefs—limit access to effective and responsive care. Although existing psychotherapeutic models have proven effective in general populations, they may not address the complex intersection of cultural identity, migration experience, and psychosocial context that shapes distress among displaced and culturally diverse groups. As a result, the risk of misdiagnosis, dropout, and ineffective treatment increases. To meet these challenges, *culturally sensitive psychotherapy* as an adaptation of therapeutic awareness and techniques, and the therapeutic relationship to be congruent with the client’s cultural context, offers a framework for integrating cultural awareness into clinical practice—not merely as a technical adaptation but as a reflective, relational, and ethical stance. This article explores culturally sensitive psychotherapy as a technique and a therapeutic attitude, drawing on models of intercultural competence and clinical examples with Turkish migrants being treated at our hospital. Our goal is to clarify how therapists can meaningfully incorporate the cultural backgrounds and differences of patients and therapists more strongly into psychotherapy.

## Key constructs

### What is culture?

The term culture is used in various disciplines in different ways or with different emphases. In general, culture may be described as the comprehensive set of behavioral patterns within a society that are transmitted symbolically across generations, expressed through tools and material products, and embedded in values and ideas (Fuchs-Heinritz et al., 1994). It also encompasses the historically and socially situated totality of both material and symbolic productions, together with internalized meanings, norms, and institutionalized ways of life (Klein, 1995). Hofstede et al. (2010) illustrate this vividly in their model of the *cultural onion*: like an onion, layer upon layer of peel is formed, with basic cultural assumptions at the innermost core, followed by values and norms, which are further encompassed by rituals, heroes and symbols. The outer layers of *symbols, rituals, and heroes* are the easiest to observe and influence, while the inner layers of values and basic assumptions are not so obvious to those unfamiliar with the culture. While the culturally relativizing *cultural onion model* refers to the description of a culture, intercultural approaches look at differences between different cultures. In an earlier model Hofstede (2001) has extracted so-called basic dimensions with his *cultural dimensions* factor analysis, which are to be regarded as descriptive categories of different cultures. The dimensions of this model are: *power distance, individualism–collectivism, masculinity-femininity, uncertainty avoidance, long-term orientation—short-term orientation, and enjoyment-restraint.* According to Hofstede, to be able to organize itself, every society is confronted with fundamental questions that are distributed across the dimensions described. Critics of the model, however, see an oversimplification of the complex phenomenon of culture based on outdated data and do not consider the complexity of multisociocultural dynamics and that the model neglects intra-cultural variability, transnational identities, and the fluid, context-dependent nature of cultural affiliation in a globalized world (e.g., McSweeney, 2002; Ailon, 2008; Fang, 2012). In general, models for a relativizing and differentiating view of culture have always been critically discussed in academic discourse, and their content-related and methodological problems have been named (e.g., Seipel and Rippl, 2013); however, they can also be understood and used as a basis for further examination of the construct of culture without disregarding the dynamic development processes of societies. The guidelines stemming from the respective cultural society are intended to guide the individual to behave according to the (religious) norms, values, expectations, and rules of their socio-cultural environment. The psychoanalyst Sigmund Freud (1930) saw culture as a *collective effort* to master external nature and regulate relationships between people. From an individual psychological perspective, Freud identifies specific difficulties that arise from the tension between individuality and culture by requiring individuals to restrain their personal desires and accept significant sacrifices in order to secure collective survival. He further argued that individuals inevitably come into conflict with cultural demands and may even resist them, which is why culture requires a degree of coercion to establish a framework that guides socialization and poses developmental challenges. The psychologist Kurt Lewin went one step further, formulating a dynamic model of individual and social behavior with his *field theory* (Lewin, 1963/2012). According to this model, a person’s goal-oriented behavior is a function of the *living space* given for a specific point in time. The latter includes the person as well as certain environmental characteristics that, depending on their weighting as *vector forces*, direct the basic energies of a need and determine the resulting behavior. In the sense of this fundamental theory, which is also regarded as a precursor to cognitive theories, culture with its manifold influences on the individual can also be understood as a vector force that significantly determines the *field* and thus the person’s behavior. Two of the aspects mentioned, culture as a socializing developmental task and the identity formation process through integration and demarcation, can also be of direct relevance in psychotherapy if disturbances in these processes lead to pathological psychological suffering. From a psychodynamic perspective, specific conflict constellations or even structural disorders can develop, such as individuation-dependency conflicts, control-submission conflicts, self-sufficiency-sufficiency conflicts, self-esteem and guilt conflicts and identity conflicts or ego-structural disorders in the dimensions of perception, control, emotional communication and attachment about the self or other people (see OPD Working Group, 2023). From a behavioral therapy perspective, as the second side of the coin, maladaptive cultural adaptation can lead to the development of dysfunctional cognitive schemata and basic assumptions, which can cause various functional impairments and symptoms. If cultural aspects can be identified in the development or maintenance of these disorders in psychotherapy, they should be taken into account and included in treatment accordingly.

### Migration and acculturation

In addition to helping people fit into a certain society or group, cultural socialization also serves to distinguish them from other groups through internalized cultural values, norms, and symbols. This process of differentiation strengthens the cultural identity of a group or even an individual. Migrants, i.e., people who leave their homes for a significant period, are confronted with specific challenges as they find themselves in an intercultural or transcultural *in-between space* between different cultures. Flight as a form of migration, in which people leave their home country and cross an international border to escape armed conflict or persecution due to their religion, nationality, membership of a particular social group, or political beliefs, is a major complicating factor in finding their way in a new (cultural) environment. Refugees, in particular, have a high prevalence of mental illness as a result of the relationship between stress and protective factors. Before the act of migration, potentially traumatic experiences and reasons for fleeing (according to Chen et al., 2017, refugees experienced 1.5 to 15.2 traumatic experiences before fleeing) are countered by subjective voluntariness and sufficient preparation time for migration as protective factors (Kizilhan and Klett, 2025). Subsequently, the flight process itself can be traumatic, e.g., due to imprisonment, assaults, or traveling under life-threatening circumstances, as well as the experience of other refugees dying on their journey together. Once they have arrived in their country of refuge, some refugees experience a sense of calm and security, and psychological symptoms often improve in the short term during this phase, which is also referred to as the *honeymoon effect of migration* (Sluzki, 2010). The further development then also depends on the residence status—while a secure residence with opportunities to work, acquire language skills, and contact with people from the home country has a favorable prognostic effect, an insecure residence status increases the experience of continued threat, sometimes with intrusive experiences of anticipated traumatization in the home country upon possible return, including suicidal impulses. Everyday stressors, such as the housing situation in shelters, limited access to social participation, and worries about those left behind, can exacerbate symptoms (including those that were previously only subclinical) and lead to the manifestation of mental illness. While secure residence status enables long-term adaptation, refugees living insecurely in the country of refuge lack the psychological valences to successfully come to terms with their new culture. The process of coming into contact and engaging with the culture of the host country in interaction with the culture of origin is referred to as acculturation. According to Berry (1997), four strategies of acculturation are distinguished, which depend on whether the migrants or their group wish to retain their own culture, whether there is contact with the host society and the circumstances of the migration, the migrant group and the host society:

1. *Segregation or separation:* the minority retains its own culture and avoids any contact with the host society. Cultural isolation is sought, and there is rejection of the dominant culture or vice versa.
2. *Integration:* in this case, elements of one’s own culture are retained, while at the same time there is contact with the host society. Both groups strive for multiculturalism, and mutual influence can occur.
3. *Assimilation:* this involves giving up one’s own culture in favor of the dominant culture while maintaining contact with the majority. The process leads to fusion with the dominant culture.
4. *Marginalization or exclusion:* this involves giving up one’s own culture without having contact with the majority. This form often occurs after cultural or ethnic uprooting.

Berry’s (1997) acculturation model, which highlights strategies such as integration, assimilation, separation, and marginalization, remains a seminal framework for understanding cultural adaptation. However, as migration patterns and sociocultural dynamics have evolved significantly over the past five decades, recent research highlights the need to revisit and expand this model. Contemporary studies emphasize the complexity, fluidity, and multidimensionality of acculturation processes in a globalized world characterized by transnational ties, hybrid identities, and digital connectivity (Schwartz et al., 2010; Sam and Berry, 2010). These developments suggest that acculturation is less a linear progression, and more an ongoing, dynamic negotiation shaped by individual, social, and structural factors beyond those originally considered by Berry (1997). The acculturation process itself can be a considerable psychological burden. This acculturation stress can lead to an identity crisis, which is even more profound the more foreign the new culture is. At the same time, social bonds, such as friendships, work, and sometimes even family bonds, are often lost. Machleidt (2007) regards migration as a further phase of individuation and refers to it in a psychodynamic sense as *cultural adolescence* or *birth as a citizen of the world*. In this phase, one’s own identity and values are fundamentally questioned and must be redefined. The development during this time can be compared to a kind of puberty. Similarly to puberty, strong emotions and feelings arise, as well as phases of superiority and the pain of separation from childhood or home. There are also existential fears of failure. Increased vulnerability can arise, particularly when discrimination, social exclusion, and isolation are experienced. On the other hand, successful acculturation can also allow for a broader horizon of experience, a multicultural orientation, and the development of dynamic and multiple identities. Overall, migration is a particularly compelling example of the necessity and principles of culturally sensitive psychotherapy, which can contribute to the overcoming of the developmental tasks of acculturation. It focuses on the complex interplay of cultural identity, trauma, and adaptation and illustrates how psychological suffering is shaped not only by individual biography but also by sociocultural transitions. This makes migration an exemplary context in which uncertainties are acknowledged, openness is promoted, and patients are supported in coping with processes of change and the redefinition of their identity.

### Specific burdens and disease patterns

Explanations for the development and maintenance of illnesses are always subjective, i.e., each person interprets the occurrence of illnesses against the background of their individual knowledge and attribution schemes. The latter are also significantly influenced by culture and determine the meaning and treatment of illness. The experiences with patients of Turkish origin in Germany, for example, show that these often assume that external factors are the cause of their illness, and religious and magical ideas can also be relevant, as in Islam, for example, illness can be seen as God’s will or fateful (Ostermann, 1990). Attempts at magical explanations can also legitimize the sick role vis-à-vis the environment (Becker et al., 1998). An example concept of magical ideas is that of *Nazar*, the *evil eye*, which can cause headaches, dizziness, nausea, psychological symptoms, states of confusion and personality abnormalities, certain physical complaints, or the severing of social relationships (Assion, 2004). A similar concept is behind *Büyü*, *the white* and *the black magic* and the idea of evil spirits or demons (*cinler*). Such concepts of illness are often accompanied by a preference for traditional healing methods or folk medicine (Becker et al., 1998). Similarly, the presentation of psychological symptoms varies across cultures and is not expressed in a culturally neutral manner; rather, their presentation, interpretation, and meaning are deeply influenced by cultural context (Kirmayer and Young, 1998; Lewis-Fernández et al., 2020). For example, depressive symptoms in Western cultures are often expressed through emotional and cognitive complaints, such as feelings of sadness, hopelessness, and worthlessness. In contrast, patients from East Asian cultures may be more likely to present with somatic complaints such as fatigue, insomnia, or headaches, with minimal reference to emotional distress (Ryder et al., 2008). Patients of Turkish origin often report somatic symptoms—*suffering is primarily experienced on the body*—and altered perceptions of the environment (Myriam and Van Moffaert, 1998; Berg, 1998). While Turkish individuals show both somatic and psychological stress responses equally, Germans display psychological reactions more frequently (Özelsel, 1990). Overall, somatization appears to be more common among Turkish patients, whereas Germans tend to exhibit more mental symptoms under stress. Higher education among Turkish migrants is associated with fewer reported symptoms (Mewes and Rief, 2009). Chronic pain studies highlight additional cultural influences in Turkish migrants: poor adaptation to German society, inactivity, female gender, and uncritical use predict greater pain-related impairment (Erim and Glier, 2007). Health care utilization is higher among Turkish migrants, particularly for cardiovascular, musculoskeletal, gastrointestinal, psychological, and psychosomatic conditions (Bilgin et al., 2003; Köpp and Rohner, 1993; Günay and Haag, 1990). Depressive disorders are also more prevalent compared to other EU migrant groups (Levecque et al., 2007). More in detail, several studies have shown a particularly high prevalence of depressive disorders among Turkish immigrants in Germany compared to both native Germans and other immigrant groups. Data from the Gutenberg Health Study indicated that first-generation Turkish migrants exhibited significantly higher rates of depression (OR 1.24, 95% CI 1.01–1.52) and suicidal ideation (OR 3.02, 95% CI 1.80–5.04) compared to native Germans (Beutel et al., 2016). Turkish migrants also showed higher odds of depressive symptoms than Polish migrants (OR 2.61) and panic symptoms (OR 3.38) (Beutel et al., 2016). In another German study, the prevalence of depression among Turkish migrants was 22.4%, considerably higher than among Polish migrants (9.6%) and native Germans (6.8%), with Turkish women being particularly affected (35.1% vs. 14.8% in men) (Beutel et al., 2016). These findings are consistent with cross-national studies across northwest Europe, which consistently identify Turkish immigrants as the migrant group with the highest prevalence of depression compared to other groups such as Moroccan migrants or native populations (Levecque et al., 2007; Missinne and Bracke, 2012).

Language, as an expressive and therapeutic medium, reflects cultural imprints and may contribute to diagnostic challenges (Knipper and Bilgin, 2010). As shown in Table 1, somatic descriptions of Turkish migrants often require a *translation* into western diagnostic frameworks to ensure accurate assessment and prevent misdiagnosis (Heim, 2004; Yildirim-Fahlbusch, 2003; Zielke-Nadkarni, 1999). Establishing a shared language between patient and therapist is therefore essential.

**Table 1:** Examples Turkish idioms of distress and disease and their psychopathological meanings (literally translated).

Turkish expression	Organ-related expression	Meaning
Kafayı üşüttüm	I have a head cold.	I am loosing my mind.
Kafayı yedim	I ate my head.	I am loosing my mind
İçim yanıyor	My insides are on fire.	I am thirsty/I am very sad (denotes deep sadness).
İçim rahatladı	My inner self is calmed.	I am reassured.
Ciğerim/Ciğerlerim yanıyor	My lungs are burning.	I am very sad (denotes deep sadness).
Burnumda tütüyor	She/he/it smokes in my nose.	I miss her/him/it very much
Burnumun direği sızladı	The column of my nose hurts.	I felt deep sadness.
Gözlerim doldu	My eyes are full.	I have tears in my eyes.
Dilim/Ağzım yandı	My tongue/mouth is burnt.	I have had a difficult/bad experience.
Boğazım düğümlendi	I have knots in my throat.	I wasn’t able to say anything.
Başımın etini yedi	She/he ate my head.	She/he babbled at me (no indication of delusions, as is often assumed).
Kalbim sıkışıyor	My heart gets tight.	I have a feeling of tightness in my chest (not necessarily an indication of organic disease).
Göğsüm daralıyor	My chest gets tight.	I have a tight feeling in my chest (not necessarily an indication of organic disease).
Kalbimi/Ciğerimi deldi geçti	It has pierced my liver/my heart.	It hurt me a lot (denotes deep sadness).
İçim şişti	My insides are inflated.	She/he babbled at me (no indication of delusions, as is often assumed).
Neredeyse patlayacağım	I’m about to burst.	I have eaten a little too much/I am very overworked (not an indication of delusions, as is often assumed).
Ödüm patladı	My bile has burst.	I was very scared.
Dilimi yuttum	I swallowed my tongue.	I was startled.
Midem yanıyor	My stomach is burning.	I have a stomach ulcer/I have heartburn.
Kollarım koptu	My arms are torn off.	I have been carrying too heavy a load/I have severe pain in my arms.
Belim koptu	My back is torn off.	I was carrying a load that was too heavy/I have severe pain in my back.
Omuzlarım çöktü	My shoulders have collapsed.	I have a lot of responsibility.
Elim Ayağım tutmuyor	My arms and feet do not hold.	I have no strength/energy.
Elim/Ayağım karıncalanıyor	I have ants in my hands and feet.	I have a tingling sensation in my hands and feet (not an indication of delusions, as is often assumed).
Dizlerimin bağı çözüldü	My knee ligaments have come loose.	I ran out of strength/energy (no indication of delusions, as is often assumed).
Gözlerimde fer kalmadı	I no longer have any light in my eyes.	I have no more energy/hope.
Beynim durdu	My brain has come to a standstill.	I cannot think any more.

The role of language in psychotherapy has been increasingly recognized, particularly in the work with bilingual or multilingual patients (Martinovic and Altarriba, 2012). Language is not merely a tool for conveying information, but actively shapes emotional expression, access to memories, and the meaning-making process. In therapeutic encounters, the choice of language can therefore influence not only the articulation of symptoms, including somatic expressions of distress, but also the development of the therapeutic alliance, the clinician’s case formulation, and ultimately treatment planning. In the context of the present discussion, the culturally shaped somatic idioms observed are relevant not only for diagnostic considerations but also for the broader clinical process, including the negotiation of shared understanding between the clinician and the patient. The findings by Martinovic and Altarriba (2012) underscore that in bilingual patients, the language used in therapy is closely related to emotional processing, cultural framing, and the accessibility of autobiographical memories. Language choice may therefore influence not only symptom expression, including somatic manifestations of distress, but also the clinician’s ability to accurately assess emotional states and to establish a strong therapeutic alliance. Especially in cross-cultural contexts, sensitivity to such linguistic dynamics is crucial to avoid diagnostic misunderstandings and to facilitate effective therapeutic engagement. Although the clinical patterns and idioms of distress described above are based on empirical data and practical experience with Turkish migrants in Germany, it is important to emphasize that these examples do not represent a universal model for all individuals with a migration background. Migrant populations are highly heterogeneous, shaped by distinct sociocultural, historical, and geopolitical contexts. For instance, intra-European migrants, such as French or Polish nationals, may face fewer linguistic and systemic barriers than refugees from conflict-affected regions such as Afghanistan or Nigeria, who often present with trauma-related conditions and profound acculturation stress. Recognizing this diversity is essential to avoid cultural homogenization. Therefore, the Turkish example presented here is intended as an illustrative case, not as a generalizable prototype.

## Current evidence

### Integration of culturally specific concepts

Despite the culturally determined peculiarities and differences, individual perceptions of illness are well compatible with the overarching Western models of illness. The Biopsychosocial Model of Health and Illness was developed in 1977 by the American internist and psychiatrist George L. Engel and is still one of the most internationally recognized models of illness. Engel (1977) takes an integrative medical approach that understands illness not purely mechanistically, but as a disorder of the interaction of physical, psychological, and social factors. The dynamic interactions between these factors are of causal importance for the development and progression of diseases. Thus, in the prevention, diagnosis, treatment, and rehabilitation of diseases, it is not only necessary to take into account biological factors (e.g., genetic characteristics), but also the socio-cultural (e.g., cultural affiliation) and psychological (e.g., coping strategies) characteristics of patients. As a supplement and extension, the vulnerability-stress model explains the interplay of multiple factors that can lead to stress management or adaptation or to maladaptation or illness. The vulnerability-stress model, originally established by Zubin and Spring (1977) in the context of schizophrenia, has since been extended to a broad range of psychiatric disorders, including depression, anxiety, and trauma-related conditions. Many of the factors mentioned are culturally shaped and can increase or decrease an individual’s resilience to stress. At its core, culturally sensitive psychotherapy is about recognizing and acknowledging the cultural, ethnic, and religious differences of patients and incorporating them into the therapeutic process according to the biopsychosocial perspective. This should take place between the two extremes of cultural pseudo-empathy vs. cultural ignorance (Kaeding and Süren, 2006, p. 222). On the therapist’s side, for example, the willingness to abandon one’s professional standards, identification with the victim, overprotection, or protection can be seen as indications of an overemphasis on culture. Implicit/explicit unrealistic demands, contemptuous attitudes, the omission of certain measures, and devaluation, on the other hand, can be seen as an expression of excessive personalization or denial of culture. Contact with people from other cultural backgrounds harbors an ambivalence in that, on the one hand, feelings of curiosity and enthusiasm can be triggered, but on the other hand, this challenge can also give rise to uncertainty and reticence due to the foreignness (Machleidt et al., 2018). As with conventional psychotherapy, the central aim of culturally sensitive psychotherapy is to establish a good and stable therapeutic relationship, which plays a key role in determining the course of treatment and therapeutic success (Hartung and Kosfelder, 2019). Patient needs, which guide the therapeutic process, are particularly important here. Complementary relationship design is also central here (Grawe, 1992), in which the therapeutic relationship is aligned with the patient’s motives and needs and enables the perception of aspects that serve to achieve the treatment goals. In this interaction with the patient, knowledge of intercultural characteristics such as nonverbal behavior (e.g., eye contact), hierarchy, dealing with authority and conflicts as well as gender differences is essential for a successful relationship. Real or suspected cultural differences can lead to misunderstandings in psychiatric-psychotherapeutic treatment. This can contribute to the emergence and reinforcement of cultural stereotypes such as *the North African*, *the refugee* or *the German psychotherapist*. Such stereotypes often lead to individual differences being hastily attributed to a supposed *culture* instead of taking a differentiated view of the individual with their unique motivational background and personal circumstances. This neglects deeper, possibly culture-independent explanations and runs the risk of slipping into racist stereotypes. Therefore, a patient-centred approach is essential to understand a person in its entirety, with all their worries, needs, wishes, aspirations, individual biography, hopes for the future, problems, and symptoms of illness. Cultural aspects should be taken into account, but not overemphasized. In working with patients of diverse cultural and linguistic backgrounds, language plays a central role not only in expression but also in the therapeutic relationship and clinical formulation. As Martinovic and Altarriba (2012) emphasize, for bilingual individuals, different languages can activate distinct emotional states, memories, and cultural frameworks, thus influencing how distress is verbalized and processed in therapy. However, as Knipper (2014) cautions, it is equally important to avoid reducing complex clinical phenomena to simplistic cultural attributions. He describes how the tendency to attribute, for example, somatic expressions of depression in Turkish patients solely to cultural norms of somatization can lead to stereotyping and diagnostic shortcuts that obscure the individual context, such as family dynamics, migration stressors, or personal trauma. Knipper refers to this risk as culturalization: the overemphasis of culture at the expense of person-centered, contextual understanding. A more nuanced approach requires clinicians to engage reflexively, recognizing their own cultural assumptions and maintaining an open, exploratory stance toward each patient’s unique life circumstances. Thus, while cultural and linguistic factors undoubtedly shape the way distress is communicated, they must always be within the broader personal, social and historical contexts that inform the patient’s experience. This perspective is particularly relevant when considering somatic idioms of distress in Turkish patients, as it highlights both the significance and limitations of cultural explanations in clinical practice.

## Conceptual synthesis and clinical illustration

As shown in the design of therapeutic relationships, culturally sensitive psychotherapy requires a range of intercultural skills that go beyond specific techniques and rather reflect and modify existing therapeutic knowledge and skills from an intercultural perspective. It is also important to extend the (inter)cultural perspective to practitioners, who naturally bring their cultural socialization and influences into the therapeutic relationship and the therapeutic process. Parlett’s (1991) Field Theory, rooted in Kurt Lewin’s social psychology, emphasizes the dynamic interplay between individuals and their social environment, viewing behavior and experience as emerging from complex and contextual fields. This perspective is also foundational in Gestalt psychotherapy, where the concept of the field denotes the total psychological and relational environment in which therapeutic change occurs (Perls et al., 1951; Yontef, 1993). Gestalt psychotherapy, drawing directly from Field Theory, considers experience as co-created in the therapeutic here-and-now. Applied to culturally diverse settings, this means that neither the therapist nor the client operates in isolation; rather, both are embedded in sociocultural fields that shape meaning-making and relationship dynamics. Thus, culturally sensitive psychotherapy informed by Field Theory recognizes that cultural identity, power asymmetries, and language are not static background variables but actively shape the therapeutic process. For example, the therapist’s awareness of their own cultural assumptions becomes a critical variable in the field. This aligns with the principles of cultural humility, which demand ongoing reflection, coconstruction of meaning, and attentiveness to shifting cultural contexts. Applying this perspective to the psychotherapeutic care of migrants highlights the necessity of understanding patients not as isolated entities but as embedded in shifting cultural, social, and systemic fields. Migrants face multifaceted challenges, such as cultural dislocation, language barriers, different concepts of illness, and social marginalization, that shape their psychological experience and treatment needs. Within this framework, a key question arises: Are existing psychotherapeutic modalities adequately equipped to address these complex and context-dependent needs or is there a pressing demand to integrate humanistic principles, such as empathy, unconditional positive regard, and cultural humility, in all therapies?

Although established cognitive behavioral, psychodynamic, or systemic approaches offer valuable tools, their effectiveness may be limited if they do not fully incorporate the humanistic emphasis on the therapeutic relationship and subjective experience within their social context. Integration of humanistic principles can foster greater flexibility, openness, and responsiveness in therapy, facilitating co-construction of meaning and enhancing trust-crucial factors for migrants navigating cross-cultural therapeutic encounters. Therefore, from the perspective of Field Theory, a pluralistic and integrative therapeutic approach that embeds humanistic values across modalities appears essential to meet the complex and dynamic needs of migrant patients in mental health systems. With their three-pillar model, Sue and Sue (2003) have attempted to systematize intercultural competencies under the terms *Awareness, Knowledge, Skills*. *Awareness* refers to the practitioner’s attitude of exploring and reflecting on their cultural integration (implying bicultural competence without the loss of cultural identity, see Berry, 1997) and considering its influence on the client’s perception and the therapeutic relationship. *Knowledge* refers to specific information and knowledge about the client’s cultural background and its influence on the client’s worldview. Finally, the pillar of *skills* encompasses specifically developed and adapted culturally sensitive intervention strategies and techniques. These include the ability to apply intercultural knowledge in therapeutic interactions, the use of culturally sensitive diagnostic tools, the adaptation of the treatment plan and therapeutic techniques to the patient’s cultural background, the involvement of interpreters and language mediators, dealing with one’s uncertainties and lack of knowledge, language and communication skills. In practice, the three pillars of the model are not to be understood independently or alongside each other, but are in a reciprocal relationship and exchange. In terms of content, a culturally sensitive treatment concept should cover the identification of situation- and culture-specific stressors and culture-specific factors that are perceived as stressful. In addition, a common culturally sensitive explanatory model of stress should be considered and the use of cultural resources as culturally embedded capacities such as religious faith, family cohesion, or community belonging, which may support coping and emotional recovery to maintain mental health, manage stress, and promote well-being. In addition, barriers to access to treatment must be removed, such as language and speech barriers.

To deepen the conceptual synthesis and illustrate how culturally sensitive psychotherapy unfolds in practice, the following clinical vignettes demonstrate the interplay of cultural explanatory models, therapeutic reflection, and concrete measures in treatment. They exemplify how cultural sensitivity is not reducible to techniques alone but emerges as a reflective stance that guides the use of interventions in dynamic therapeutic fields.

### Case 1

A 35-year-old man from Central Africa was admitted to an acute psychiatric ward under police escort due to florid psychosis with command hallucinations and the conviction of being possessed by an evil spirit. Due to language barriers, a professional interpreter was involved. During the exploration of hallucinations and delusional content, the interpreter suddenly broke down in tears and refused to continue, fearing that the ‘possession’ could be transferred to him.

This incident vividly illustrates how cultural explanatory models can shape not only the patient’s illness experience but also the emotional responses of professionals and interpreters, with direct consequences for diagnostic clarity and therapeutic continuity. The clinical team therefore implemented several therapeutic measures: a clinically experienced interpreter was engaged to ensure communication; team-based reflection and supervision were initiated to process fears and avoid premature cultural or religious labeling; and psychoeducation was offered to staff to strengthen their capacity for intercultural encounters. With the patient, therapeutic work focused on acknowledging his own explanation of possession while carefully introducing psychiatric concepts, thus maintaining respect for his worldview while situating symptoms in a treatment-oriented framework. These steps, interpreting, reflecting, educating, communicating, were not isolated techniques but expressions of a reflective therapeutic stance. By integrating them, clinicians were able to re-establish continuity of care, protect the therapeutic alliance, and ensure patient safety.

### Case 2

A 64-year-old first-generation Turkish migrant presented with several months of severe headaches, dizziness, and recurrent nausea. Extensive internal medical examination revealed no pathological findings. She attributed her symptoms to *Nazar* (evil eye), a cultural explanation supported by her family, who also encouraged consultation with a traditional healer (Hoca). Psychiatric evaluation revealed significant migration-related stress, including social isolation, language barriers, and a conflicting relationship with her husband. She reported sleep disturbances, loss of appetite, and feelings of hopelessness, which she considered less relevant than her somatic complaints.

Therapeutic measures in this case included consistent use of a professional interpreter to bridge linguistic and cultural gaps, integration of psychoeducation that linked her explanatory model with psychiatric understanding, and pharmacotherapy to address depressive symptoms. At the same time, the therapeutic dialogue was carefully adapted to her cultural framework: The clinician explicitly validated her interpretation of *Nazar* as a meaningful way of making sense of her suffering, while also introducing a biopsychosocial perspective that connected her somatic complaints with migration-related stress. In addition, supportive interventions were directed at mobilizing her social resources and reducing isolation. The therapeutic progress depended less on prescribing medication or delivering information alone than on maintaining an attitude of cultural humility: the clinician worked collaboratively with the patient and her family, respecting their explanatory models while creating space for new perspectives. By doing so, the treatment promoted trust, increased adherence, and allowed a more comprehensive understanding of her distress.

## Conclusion

In general, culturally sensitive psychotherapy must be understood both as a technique and as a specific attitude. In addition to specific interventions and strategies, it means a fundamental attitude toward cultural variety and diversity. A culturally sensitive attitude requires continuous reflection and self-examination on the part of the therapist. They must be aware of how their cultural backgrounds and prejudices can influence their work and be prepared to question and, if necessary, overcome them. This requires a certain flexibility and willingness to change on the part of therapists to ensure that they can support their patients in the best possible way. Ultimately, it is precisely this attitude that forms the core of culturally sensitive practice and shapes the entire therapeutic relationship. It ensures that therapists treat their patients in a respectful, empathetic, and effective manner. When uncertainties arise regarding possible cultural influences, curious and respectful inquiry should be approached from a learner’s perspective. This therapist’s stance strengthens the therapeutic relationship, opens up new avenues of exploration, and deepens the collaborative work. Last but not least, culturally sensitive psychotherapy can be used to experience similarities and differences in encounters with people from other cultures, which can enrich both the therapist’s thinking and actions and those of the patient.

While numerous frameworks describe cultural differences in behavior, communication, and distress expression (e.g., Berry’s acculturation model, Hofstede’s cultural dimensions, Engel’s biopsychosocial model), these approaches risk overgeneralization when applied too rigidly in clinical practice. Recent discussions emphasize cultural humility as a more dynamic alternative that highlights the clinician’s ongoing self-reflection, openness, and recognition of power differentials by the clinician (Hook et al., 2013; Tervalon and Murray-García, 1998). In psychiatric and psychotherapeutic settings, operationalizing cultural humility requires structured strategies that go beyond checklists of cultural competence. In this manuscript, we argue that recognition of somatic idioms of distress, as observed in Turkish patients in Germany, provides a useful clinical context to illustrate both the necessity and the challenges of implementing cultural humility within evidence-based care. Rather than assuming cultural patterns as static explanatory models, cultural humility promotes a collaborative, patient-centered approach that allows for individualized assessment, nuanced case formulation, and flexible treatment planning. Cultural humility offers a vital framework for understanding somatic complaints as legitimate and meaningful expressions of distress rather than mere signs of a disorder of emotional perception. By encouraging clinicians to approach patients with openness and a willingness to learn from their unique cultural and linguistic backgrounds, cultural humility shifts the perspective from pathologizing somatic symptoms to appreciating them as communicative acts embedded in a personal and cultural context. This helps to avoid the frequent misinterpretation of somatic expressions as medically unexplained or purely psychosomatic phenomena. Consequently, the diagnostic process benefits from this stance by facilitating a more nuanced differential diagnosis. For example, distinguishing between depression and somatoform disorders can be challenging when patients present predominantly with physical symptoms. Cultural humility encourages clinicians to explore these symptoms in dialogue with patients, considering cultural idioms of distress and individual meaning-making, leading to more accurate and context-sensitive diagnoses. Additionally, adopting cultural humility can significantly enhance the therapeutic alliance. Patients who perceive that their modes of expression, verbal, somatic, or symbolic—are acknowledged and respected are more likely to engage openly in therapy. This mutual respect fosters trust, reduces barriers related to stigma or cultural misunderstanding, and supports collaborative treatment planning that aligns with values and lived realities.

## Implications

Current recommendations for integrating cultural considerations into psychiatric and psychotherapeutic practice are often brief and generic, lacking detailed clinical tools or structured training frameworks. While frameworks such as the International Classification of Functioning, Disability and Health (ICF) and the cultural formulation sections of DSM-5 and ICD-11 represent important steps towards systematizing the assessment of cultural factors, their practical implementation remains limited. The ICF offers a comprehensive biopsychosocial model that acknowledges environmental and personal factors, but it does not provide explicit guidance on operationalizing cultural aspects during clinical encounters (Cieza and Stucki, 2008). Similarly, the Cultural Formulation Interview (CFI) of DSM-5 and ICD-11’s cultural guidelines facilitate structured cultural evaluations, but their use requires substantial clinical training and time resources that may not be readily available in everyday clinical settings (Lewis-Fernández et al., 2014; WHO, 2018). To bridge this gap, practical, accessible clinical tools are essential to support cultural humility and cultural formulation in routine care. For example, a stepwise cultural formulation worksheet can guide clinicians through key domains such as cultural identity, explanatory models of illness, psychosocial environment, and dynamics of the patient–clinician relationship (Aggarwal and Lewis-Fernández, 2020). This tool prompts reflective inquiry and fosters meaningful dialogue, enhancing both diagnostic accuracy and therapeutic engagement. In addition, clear guidelines for working with interpreters are critical given the central role of language in cross-cultural care. Best practices include briefing interpreters before sessions, managing the triadic interaction to maintain rapport, and ensuring confidentiality and cultural sensitivity throughout (Flores, 2005; Karliner et al., 2007). Integrating such training into psychiatric and psychotherapeutic education can further operationalize cultural humility. By developing and disseminating these concrete tools and educational frameworks, clinical practice can move beyond abstract recommendations toward actionable strategies that improve care for culturally diverse patients.
